# Comparison of Extraction Methods of Chitin from *Ganoderma lucidum* Mushroom Obtained in Submerged Culture

**DOI:** 10.1155/2014/169071

**Published:** 2014-01-15

**Authors:** Sandra Patricia Ospina Álvarez, David Alexander Ramírez Cadavid, Diana Marcela Escobar Sierra, Claudia Patricia Ossa Orozco, Diego Fernando Rojas Vahos, Paola Zapata Ocampo, Lucía Atehortúa

**Affiliations:** ^1^Biotechnology Group, Natural Sciences Faculty, Universidad de Antioquia, Calle 70 No. 52-21, Medellín, Antioquia, Colombia; ^2^Biomaterials and Biomechanical Research Group, Bioengineering Program, Universidad de Antioquia, Calle 67 No. 53-108, Bloque 18-227 Medellín, Colombia

## Abstract

The chitin was isolated from the *Ganoderma lucidum* submerged cultures mycelium as potential source of chitin under biotechnological processes. The extraction of chitin was carried out through 5 different assays which involved mainly three phases: pulverization of the mushroom, deproteinization of the mycelia with NaOH solution, and a process of decolorization with potassium permanganate and oxalic acid. The chitin contents extracted from 9-day mycelia were 413, 339, 87, 78, and 144 mg/g^−1^ (milligrams of chitin/grams of dry biomass) for A1, A2, A3, A4, and A5, respectively. Obtained chitin was characterized by X-Ray Diffraction (XRD), by Fourier transform infrared spectroscopy (FTIR), and by thermal analysis (TGA). The results showed that *Ganoderma lucidum* chitin has similar characteristic of chitin from different fonts. The advantage of the biotechnological processes and the fact that *Ganoderma lucidum* fungus may be used as a potential raw material for chitin production were demonstrated.

## 1. Introduction

Chitin is the second most abundant biopolymer in nature found in shells of crustaceans, in the cuticles of insects, and in fungi cell walls. Chitin fungi cell walls such as mushrooms are a linear-chain polymer composed by 1,4-linked 2-acetamido-2-deoxy-*β*-D-glucopyranose units and classified as *γ*-chitin [1–4]. Chitin and its derivative—chitosan—are natural aminopolysaccharides, which have unique structures, multidimensional properties, highly sophisticated functions, and a wide range of applications in biomedical areas and other industrial areas [1, 5, 6]. The most important characteristics of this biomaterial are excellent biocompatibility and biodegradability with ecological safety and low toxicity. In addition, chitin and chitosan have versatile biological activities and low immunogenicity [5]. Chitosan has become of great interest as a new functional biomaterial of high potential in various fields such as pharmaceutical, medical, cosmetic, feeding, and agricultural ones [7–10].

The most common source of chitin comes from shells of crabs, shrimps, and krills which are wastes from the processing of marine products. The annual worldwide crustacean shells production has been estimated in 1.2 × 10^6^ tons, and the recovery of chitin and protein from these crustacean wastes is an additional source of revenue [11]. However, crustacean shell wastes can be limited and subject to seasonal supply. In recent years, chitin obtained by extraction from fungi mycelia is gaining importance. Fungi mycelia can be cultivated throughout the year by fermentation under submerged culture which is rapid and synchronized and can be performed in bioreactors with all automated and controlled conditions; therefore, mycelial biomass produced in each batch is homogeneous, in quality and quantity [6, 12].

In this study, five protocols for isolating chitin from *Ganoderma lucidum* produced under submerged culture conditions, in an optimized culture medium for biomass production, have been reported [12]. Chitin analysis was performed using X-ray Diffraction, Fourier Transform Infrared spectroscopy and thermal analysis. The chitin content was also determined.

## 2. Experimental

### 2.1. Reagents

All of the reagents used were of high purity grade. Sodium hydroxide, potassium permanganate (KMnO_4_), oxalic acid (C_2_H_2_O_4_), and ethanol 96% were obtained from Merck KGaA (Darmstadt, Germany).

### 2.2. Microorganism

Strain of *Ganoderma lucidum* was grown at 24°C for 9 days on potato dextrose agar (PDA) and was periodically transferred onto a new PDA medium. The strain was maintained at 4°C.

### 2.3. Culture

Actively growing mycelia were obtained from a newly prepared agar-plate culture after being incubated for 9 days at 24°C. The preinoculums were prepared as follows: about 1 cm × 1 cm of mycelia was inoculated into a 250 mL erlenmeyer flask, which contained 50 mL of complex medium [12] (50 g L^−1^ barley flour, 0.03 g L^−1^ K_2_HPO_4_, 0.08 g L^−1^ NaNO_3_, 0.01 g L^−1^ KCl, and 0.02 g L^−1^ MgSO_4_·7H_2_O). Flasks with cultivated mycelium were placed in a shaker at 100 rpm for 7 days.

Subsequently, a bioreactor (Bioflo 110 Reactor New Brunswick with 5 L of work volume) was inoculated with 400 mL of *Ganoderma lucidum* submerged culture and the culture was grown with an agitation rate of 200 ± 2 rpm and an aeration of 5 vvm (L/L/min, means in 1 minute time there is 5 liters of air passing through 1 liter of culture medium). All cultures were carried out at temperature of 26 ± 1°C in bioreactor and pH 6.0 for 14 days.

### 2.4. Biomass and Substrate Quantification from *Ganoderma lucidum* Cultures

*Ganoderma lucidum* growth kinetics was followed during the time of culture; 100 mL of sample were taken out from the bioreactor every day during culture time. The sample was filtered through a US standard mesh number 35-screen sieve and washed twice with deionized water. Fungi biomass was dried by lyophilization (FDU-100 Lyophilizer Eyela) in order to obtain and measure the amount of mycelium formed; that is, the parameter used for growth kinetics was dried biomass weight. All conditions were monitored throughout the culture period and the experiment was performed in triplicate.

### 2.5. Chitin Isolation

Chitin was prepared from *Ganoderma lucidum* according to three modified protocols of Synowiecki and Al-Khateeb [13], Yen and Mau [6] and Su et al. [14]. Five assays for the chitin isolation were made as follows: for the first 3 assays, the dried fungi biomass was pulverized and was subjected to alkaline treatments with sodium hydroxide solution (NaOH) at a ratio of 1 : 30 (w/v). For each assay, molar concentrations, temperature, and reaction time were varied; for A1 assay: 1 M NaOH 1 : 30 (w/v), 40°C for 2 h; for A2 assay: 1 M and 2 M NaOH 1 : 30 (w/v), 90°C for 2 h; and finally for A3 assay: 2 M and 4 M NaOH 1 : 30 (w/v), 90°C for 2 h. In A3 assay, a decolorization process was added; the crude chitin was treated with 10 g L^−1^ potassium permanganate for 1 h and then reacted with 10 g L^−1^ oxalic acid for 1 h.

Assays A4 and A5 had different processes. In A4 assay, the dried fungi biomass was pulverized and a part of it was subjected to extraction twice with hot water for removing some unwanted polysaccharides. The residue was collected and dried in an oven at 40°C. Deproteinization was performed using alkaline treatment with different molar concentrations of NaOH (2, 4, 6, and 8 M) in 1 : 20 (solid : alkali) ratio, at 100°C for 3 h. The suspension was centrifuged and washed with deionized water until reaching neutrality. Decolorization process was similar to the one carried out in A3 assay.

In A5 assay, the dried fungi biomass was pulverized and mixed with deionized water, and then the mixture was subjected to a sonication process for 40 minutes and then centrifuged. The powder was washed with ethanol for 24 h. Deproteinization was performed using alkaline treatment with 4 M NaOH at the ratio of 1 : 20 (w/v), at 100°C for 2 h. This treatment was repeated three times. The suspension was centrifuged and washed with deionized water until reaching neutrality. Decolorization process was similar to the one carried out in A3 assay.

At the end of the processes of each assay, each suspension was centrifuged and washed with deionized water until reaching neutrality and dried at 50°C until reaching constant weight. Finally, the amount of chitin produced by each protocol was determined by dried weight method.

The conditions such acid and basic concentrations, time, and temperature of reactions of the five assays are presented in Table 1.

### 2.6. Chitin Characterization

The X-ray diffraction (XRD) analysis was applied to detect the crystallinity of prepared chitins and their patterns were recorded using a X'Pert PRO MPD diffractometer from PANalytical with Cu K*α* radiation of 1.5406 Å, power of 1.8 KW (40 mA, and 45 KV). Data were collected at a scan rate of 2°/min with the scan angle from 3 to 50°.

Fourier transform infrared spectroscopy (FT-IR) analysis was applied to samples with better crystallinity; that is, this analysis was used to analyze the molecular structure of isolated chitin in A5 and their spectrum was recorded using a SpectrumOne Spectrophotometer Perkin Elmer. The average number of scans taken per sample was 8 in the spectral region, between 450 and 4000 cm^−1^, with a resolution of 4 cm^−1^. The samples were prepared in 0.25 mm thickness KBr pellets (5 mg in 100 mg of KBr) and stabilized under controlled relative humidity before obtaining the spectrum. For developed preliminary protocols, corresponding to Assays A1, A2, A3, and A4, it was decided to analyze the main peak corresponding for chitin located at 2*θ* = 19° through X-ray diffraction. Using the results obtained by this technique, the crystallinity of every sample was analyzed and it was decided to carry out infrared analysis to the more crystalline sample (A5). For this reason, the infrared spectra are not presented.

TGA assay was conducted with an Instrument TGA Q500. The sample was heated until 900°C at 10°C/min, in air atmosphere.

## 3. Results and Discussion

### 3.1. Biomass and Substrate Quantification from *Ganoderma lucidum* Cultures

**Figure 1:**
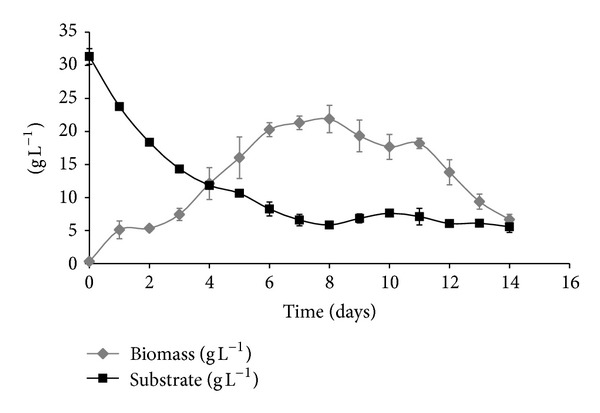
Biomass production and substrate uptake kinetics of *Ganoderma lucidum* growing in liquid culture in a New Brunswick Bioflo 110 Reactor of 5 L.

Figure 1 shows the growth kinetics for biomass production and substrate uptake for 14 days of culture.

In Figure 1, the different phases of growth during the culture, such as lag phase (day 0–2), log phase (day 2–6), stationary phase (day 2–8), and death phase (day 8–14), are shown. Maximum biomass production was identified at 8th-day with an amount of 21.87 ± 2.2 g L^−1^. Likewise substrate uptake kinetics was identified for 14 days of culture; substrate decreased during the days of culture until a minimum of 5.57 ± 0.85 g L^−1^. The last substrate uptake curve maximum was registered in the first 6 days, after that, decreasing was constant until the last day of culture.

Due to the growing scientific and technological interest in increasing mycelial biomass production of *Ganoderma lucidum* to overcome production problems in solid fermentation and improve values obtained in liquid fermentation or under submerged culture conditions, our research group has sought alternatives for increasing production of biomass and metabolites of interest.

The highest biomass yield achieved was 21.87 ± 2.2 g L^−1^. This biomass yield was higher than those obtained by Berovič et al. [15] and Tang and Zhong [16], who used carbon sources such as glucose and lactose, respectively. However, the biomass yield was similar to the biomass obtained by Tang et al. [17].

### 3.2. Chitin Isolation

Chitin was obtained from *Ganoderma lucidum* dried biomass by means of alkaline treatment, acid treatment, and/or decolorization processes with potassium permanganate, and oxalic acid The amount of chitin obtained was between 78 and 413 mg g^−1^ in different assays (milligrams of chitin for grams of dry biomass). *Ganoderma lucidum* chitin yield percentages are shown in Table 2. It is possible to observe that chitin production amounts are different. A1 and A2 show the highest amounts of chitin production. A difference between A1 and A2, with A3, A4, and A5 was the decolorization treatment with potassium permanganate (KMnO_4_) and oxalic acid (C_2_H_2_O_4_); A3, A4, and A5, showed low chitin yield likely due to the strong oxidant nature of potassium permanganate. It is possible that the treatment has not only removed pigments but chitin also. A similar result was obtained by Yen and Mau [6].

Besides in A4, the lowest chitin yield was shown—7.8% chitin—, possibly due to the fact that it was prepared with hot water and decolorization treatment. Polysaccharides were eliminated through the use of hot water, thus creating a probability that chitin obtained is purer than the one obtained in other assays. It is therefore important to carry out a removal treatment of fungi polysaccharides in order to obtain pure chitin. In addition, in A5, an ethanol treatment, which could precipitate polysaccharides, was carried out for obtaining a purer chitin [22].

### 3.3. Chitin Characterization

**Figure 2:**
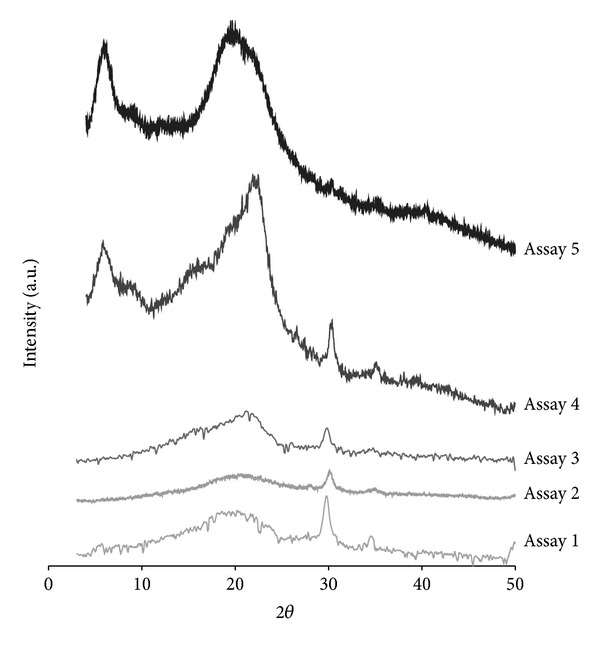
X-ray chitin assays 1 to 5 diffraction patterns.

The best results of XRD analysis for each assay are shown in Figure 2. Diffractograms for the first three assays (A1, A2, and A3) exhibit one broad peak, approximately located at 2*θ* = 18°–21° and another at 2*θ* = 30°. A4 chitin XRD pattern shows three crystalline peaks at 2*θ* = 5.6°, 21.7°, and 30.1°; while A5 XRD pattern shows high intensity peaks at 2*θ* = 5.7°, and 19.6°.

A1, A2, and A3 XRD patterns shown in Figure 2 were similar; however, A2 XRD pattern had peaks with the lowest intensity. They are similar to the ones reported by Yen and Mau [6] diffractograms of A4 and A5 presented a marked increase in the intensity of crystalline peaks. Apparently, concentrations of alkaline solutions did not affect chitin crystallinity obtained; it was observed that the decolorization process implemented in A3, A4, and A5 increased the crystallinity.

The peak approximately located at 2*θ* = 30° present in the four first assays is not characteristic of chitin. A5 XRD pattern shows high intensity peaks corresponding to chitin pattern. Reviewing XRD patterns of the reagents used for extraction of chitin, it was observed that corresponds to characteristic peak of NaOH, indicating that there was not a complete elimination of this compound and it still remained in small amounts in the analyzed samples. These results suggest the presence of chitin with one or more undesirable compounds, such as compounds not eliminated from fungi biomass or waste from reagents used in the chitin obtaining process; which produce a broad peak near 2*θ* = 19° and non-characteristic peaks of the interested biopolymer. Some authors have reported characteristic crystalline peaks of the different types of chitin which are shown in Table 3.

The results seem to confirm that the chitin obtained in the five assays shown above has the characteristic peak of standard chitin, about 2*θ* = 19°. Besides, the chitin obtained at A4 and A5 showed the characteristic peak of standard chitin, around 2*θ* = 5.7°. However, the assay A5 showed the better DRX spectrum, possibly due to the three consecutive deproteinization, made and to a sonication process which help to break fungal biomass cell membranes and makes reaction easier.

The two characteristic crystalline peaks with slightly fluctuated diffraction angles found in the XRD patterns indicated that two types of *α*- and *γ*-chitin had two consistent peaks of 2*θ* = 9°-10° and 19°-20° [23].

**Figure 3:**
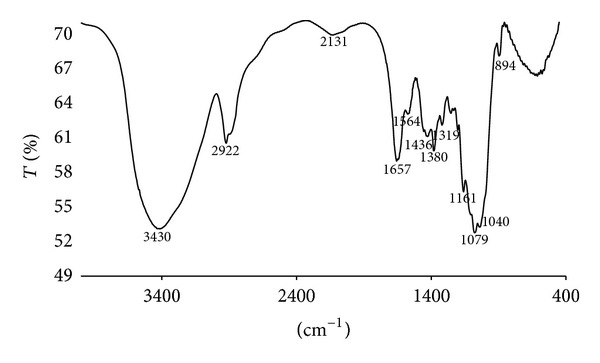
IR spectrum for chitin (A5).

FTIR analysis was made for chitin from A5 and it is shown in Figure 3. The FTIR shows chitin representative bands at 3,430, 2,922, 2,131, 1,657, 1,564, 1,380, 1,319, 1,161, 1,079, and 894 cm^−1^.

A5 FTIR analysis (Figure 3) shows chitin representative bands according to report by Paulino et al. [24] and Brugnerotto et al. [25]. The spectrum reveals the presence of a broad band at 3,430 cm^−1^, which corresponds to the vibrational stretching of the hydroxyl groups, consistent with those reported by Paulino et al. [24] and Fernández et al. [26].

The band at 2,922 cm^−1^ represents the C–H a symmetric stretching. Teng et al. [27] reported that bands near to 2,900 cm^−1^ are representative bands for chitin. The band at 1,657 cm^−1^ corresponds to the amide I stretching of C=O, while the band at 1,564 cm^−1^ corresponds to the stretching or N–H deformation of amide II, and bands at 1,380 cm^−1^ and 894 cm^−1^ correspond to the symmetrical deformation of amide III.

Additionally, FTIR spectrum between chitin from *Ganoderma lucidum* of the final assay and standard chitin from crab shells, Sigma-Aldrich (spectrum to the standard chitin not shown in this paper) presented a correlation percentage of 79.53%. XRD results for this assay showed a pattern of peaks corresponding to standard chitin peaks at 2*θ* = 5.7° and 19.6°. From previous results, it is possible to state that chitin from this fungus may be a promising source to obtain this biomaterial.

**Figure 4:**
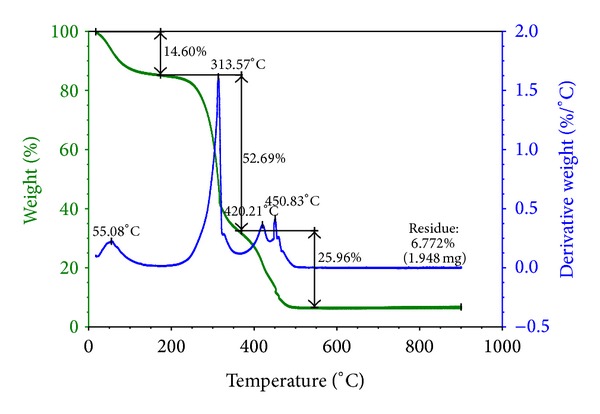
TGA for chitin (A5).

The thermogravimetric analysis of chitin A5 assay is shown in Figure 4, which presented peaks at 55.08, 313.57, 420.24, and 450.83°C. It was could be observed that after 500°C the percentage of residual mass remained constant and it was low (6.772%).

In the chitin thermogram (Figure 4), three decomposition steps are shown. In the interval between 0 and 100°C, the peak is attributed to water evaporation. The peak at 313.57°C could be attributed to the degradation of the saccharide structure of the molecule, including the dehydration of saccharide rings and the polymerization and decomposition of the acetylated and desacetylated units of chitin, as reported by Paulino et al. [24]. The third step occurred in the range of 400–500°C due to complete degradation of the polymer.

In similar TGA and DTG thermal analysis tests, other researchers found different results for chitin obtained from other sources. A DTG peak at 400°C in chitin produced from silkworm chrysalides was reported by Paulino et al. [24] Nogi et al. [28] extracted chitin from fresh shells of black tiger prawn (*Penaeus monodon*); the chitin showed higher thermal stability and began to degrade from around 280°C, and their DTG peak was observed at 400°C. Stolarek and Ledakowicz [29] isolated chitin from arctic krill scuta (*Euphasia superba*) and studied its descomposition kinetics; their results showed that a maximum rate of chitin decomposition was observed in a higher temperature range (350–380°C). The chitin obtained from *Ganoderma lucidum* fungus has lower thermal stability since the degradation starts at 313.57°C; however in a similar result were obtained by Yen and Mau [6], who used DSC analysis, it was observed that the degradation of chitin from shiitake stipes occurred approximately at 248°C.

As mentioned above, crustaceans are commonly used for both the production of chitin and the elaboration of new procedures for its isolation. At the same time, fungi as a source of chitin possess a number of advantages, namely, high growth rate and low content of minerals, and there is the possibility that their waste can be used in both food and paper industries. Since chitin predominates in the morphogenesis of fungi, the development of isolation procedures and the study of chitin-containing materials have become of great importance [30].

Extraction of chitin from fungi provides a more controllable route for obtaining a purer and more consistent chitin than the one obtained from shellfish waste. Furthermore, chitin extracted from fungi is less likely to have allergenic contaminants than chitin obtained from shells and is therefore more suitable to be used in textile, food, and pharmaceutical applications. There is a great need for identifying fungal sources which produce quantities of chitin sufficient to justify commercial production [31].

## 4. Conclusions

Biotechnological process for biomass production of *Ganoderma lucidum* is a suitable method for alternative production of chitin. This is mainly due to high biomass production from which 14.42 mg g^−1^ (milligrams of chitin/grams of dry biomass) is obtained. Therefore the biomass of this fungus is an excellent raw material for production of chitin.

The five XRD patterns were similar and presented a crystalline peak around of 2*θ* = 19°. However the A5 XRD analysis showed the highest crystallinity, with peaks in 2*θ* = 5.7° and 19.6°.

The highest production of chitin was obtained in A1 (41% g/g); nevertheless, XRD and FTIR analysis showed that chitin obtained at A5 was more similar to the standard chitin. Therefore, best results were obtained when the biomass was sonicated and washed with ethanol, deproteinized with 4 M NaOH at 100°C for 2 h, and then bleached using potassium permanganate and oxalic acid.

The chitin obtained from *Ganoderma lucidum* fungus has lower thermal stability than chitin obtained from other common sources such as shells of crustaceans; however it is thermally more stable than chitin obtained from another mushroom.

Although there are several papers in which the chitin and chitosan obtaining process is shown, in the literature consulted, nothing about chitin obtained from *Ganoderma lucidum* mushroom was found. This paper presents research results currently innovative, because all the technique used for processing and material manufacturing chitin correspond to chitin extracted from crab shells.

Finally, it can be concluded that the biomass from *Ganoderma lucidum* obtained by biotechnological culture is a promising source for obtaining chitin and its by-products.

## Figures and Tables

**Table 1:** Conditions for the chitin isolation.

Assay	Treatment 1	Treatment 2	Treatment 3
A1	Dried and powdered biomass	Deproteinization NaOH 1 M 1 : 30 (w/v) 40°C, 2 h	
A2	Dried and powdered biomass	Deproteinization NaOH 1 M, 2 M 1 : 30 (w/v) 90°C, 2 h	
A3	Dried and powdered biomass	Deproteinization NaOH 2 M, 4 M 1 : 30 (w/v) 90°C, 2 h	Decolorization KMnO_4_ 10 g L^−1^, 1 h C_2_H_2_O_4_ 10 g L^−1^, 1 h
A4	Dried and powdered biomass, immersion in hot water in order to remove polysaccharides	Deproteinization NaOH 2 M, 4 M, 6 M, and 8 M 1 : 20 (w/v) 100°C, 3 h	Decolorization KMnO_4_ 10 g L^−1^, 1 h C_2_H_2_O_4_ 10 g L^−1^, 1 h
A5	Dried and powdered biomass and sonication 40 min washed with ethanol 24 h	Deproteinization NaOH 4 M 1 : 20 (w/v) 100°C, 3 h Three times.	Decolorization KMnO_4_ 10 g L^−1^, 1 h C_2_H_2_O_4_ 10 g L^−1^, 1 h

**Table 2:** Chitin yield percent of obtained from *Ganoderma lucidum* mycelial biomass.

Assay	Yield chitin (%)
A1	41
A2	34
A3	9
A4	8
A5	14

**Table 3:** Characteristic peaks of X-ray diffraction of *α*-, *β*-, and *γ*-chitin reported by some authors.

Author	Type of chitin	Characteristic peaks 2*θ*	Source
Jang et al. [18]	*α*-chitin	9.6°, 19.6°, 21.1°, and 23.7°	Crab shell
	*β*-chitin	9.1° and 20.3°	Squid pen
	*γ*-chitin	9.6° and 19.8°	*Lucainade *
Cárdenas et al. [19]	*α*-chitin	19.18° and 19.26°	Shrimp, prawn, king crabs, and lobster
	*β*-chitin	18.78°	Squid
Kim et al. [20]	*β*-chitin	9.8° and 19.3°	Squid
Yen and Mau [21]		9.3° and 19°	Crabs
Yen and Mau [6]		5.4°–5.6°, 9.1°, and 19.3°–19.6°	*Lentinula edodes *
